# *BRAF* mutations may identify a clinically distinct subset of glioblastoma

**DOI:** 10.1038/s41598-021-99278-w

**Published:** 2021-10-08

**Authors:** Samantha N. McNulty, Katherine E. Schwetye, Cole Ferguson, Chad E. Storer, George Ansstas, Albert H. Kim, David H. Gutmann, Joshua B. Rubin, Richard D. Head, Sonika Dahiya

**Affiliations:** 1 Department of Pathology and Immunology, Washington University School of Medicine, 660 South Euclid Ave, St. Louis, MO 63110 USA; 2 Department of Genetics, Washington University School of Medicine, 660 South Euclid Ave, St. Louis, MO 63110 USA; 3 Division of Medical Oncology, Washington University School of Medicine, St. Louis, MO USA; 4 Siteman Cancer Center, Washington University School of Medicine, St. Louis, MO USA; 5 Department of Neurosurgery, Washington University School of Medicine, St. Louis, MO USA; 6 Department of Neurology, Washington University School of Medicine, St. Louis, MO USA; 7 Department of Pediatrics, Washington University School of Medicine, St. Louis, MO USA; 8 Department of Neuroscience, Washington University School of Medicine, St. Louis, MO USA

**Keywords:** Cancer, Genetics

## Abstract

Glioblastoma (GBM) is the most common primary malignant brain tumor in adults. Prior studies examining the mutational landscape of GBM revealed recurrent alterations in genes that regulate the same growth control pathways. To this regard, ~ 40% of GBM harbor *EGFR* alterations, whereas *BRAF* variants are rare. Existing data suggests that gain-of-function mutations in these genes are mutually exclusive. This study was designed to explore the clinical, pathological, and molecular differences between *EGFR-* and *BRAF-*mutated GBM. We reviewed retrospective clinical data from 89 GBM patients referred for molecular testing between November 2012 and December 2015. Differences in tumor mutational profile, location, histology, and survival outcomes were compared in patients with *EGFR-* versus *BRAF-*mutated tumors, and microarray data from The Cancer Genome Atlas was used to assess differential gene expression between the groups. Individuals with *BRAF*-mutant tumors were typically younger and survived longer relative to those with *EGFR-*mutant tumors, even in the absence of targeted treatments. *BRAF*-mutant tumors lacked distinct histomorphology but exhibited unique localization in the brain, typically arising adjacent to the lateral ventricles. Compared to *EGFR-* and *IDH1-*mutant tumors, *BRAF-*mutant tumors showed increased expression of genes related to a trophoblast-like phenotype, specifically *HLA-G* and pregnancy specific glycoproteins, that have been implicated in invasion and immune evasion. Taken together, these observations suggest a distinct clinical presentation, brain location, and gene expression profile for *BRAF*-mutant tumors. Pending further study, this may prove useful in the stratification and management of GBM.

## Introduction

Glioblastoma (WHO grade IV glioma, GBM) is a common malignancy of the central nervous system (CNS), constituting approximately 15% of all primarily brain tumors in adults^1^. The median survival rate of a GBM patient is approximately 14 months following the standard treatment of resection, irradiation, and chemotherapy^2^, a figure that has remained essentially unchanged despite significant advances in defining the molecular pathogenesis of these cancers^3^. With the dawn of the genomic era and the widespread implementation of clinical next-generation sequencing (NGS), there has been an intense search for sequence variants with diagnostic, prognostic, and/or therapeutic significance to inform optimized treatment protocols improve outcomes for GBM patients.

GBM harbor a wide array of molecular alterations, including combinations of point mutations, insertions and deletions (indels), copy number variants, and epigenetic modifications^4^. Some of these alterations correlate with differences in clinical outcome and are used to diagnose and classify tumors and/or inform treatment decisions. For example, mutations in *IDH1* and *IDH2* are the major stratifying features of diffuse gliomas^4^, and *MGMT* promoter methylation is associated with longer overall survival and response to temozolomide treatment^2^. The biological and clinical significance of other recurrent mutations has yet to be determined^4^; however, molecular data are being actively mined in search of features that could enhance the clinical stratification of GBM and reveal targets for existing and/or novel chemotherapeutic agents.

GBM frequently harbor mutations that activate mitogenic signaling pathways. Receptor tyrosine kinases (RTKs) regulate growth factor signaling to increase cell proliferation, metabolism, and survival in response to environmental cues^5,6^. Increased activation of the epidermal growth factor receptor (EGFR), a membrane bound RTK, is common in GBM. This may occur via increased protein expression or via mutations that lead to constitutive activity^7^. In a 2013 survey of 585 GBM, 45% had putative activating mutations (either copy number amplifications, exon or domain deletions, point mutations, or combinations thereof) in *EGFR*^4^. Some evidence (albeit inconsistent) suggests that gain-of-function alterations in *EGFR* could serve as an indicator of poor prognosis within the current treatment paradigm^8–10^. They are also being explored as a potential target for FDA-approved RTK inhibitors^11^.

The serine/threonine-protein kinase, B-Raf (encoded by the *BRAF* gene) is a member of the RAS/RAF/MEK/MAPK that functions downstream of EGFR. B-Raf inhibitors are approved for use in melanoma and non-small cell lung cancer^12,13^, and ongoing clinical trials are exploring their use in brain tumors (see ClinicalTrials.gov study IDs NCT03224767, NCT03973918, NCT02684058, etc.). The canonical BRAF p.V600E mutation occurs in roughly half of all epithelioid GBM^14^, but it is rare in classic GBM^15^. As such, the 585-patient cohort described above contained only 13 GBM specimens with putative gain-of-function *BRAF* amplifications and/or mutations^4^.

Activating alterations in *EGFR* and *BRAF* were reported in multiple GBM datasets, always appearing in a mutually exclusive pattern^4,16^. This observation led us to speculate that GBM with activating alterations in *BRAF* may constitute a biologically distinct entity. In this study, we sought to determine the clinical, pathologic, and molecular differences between GBM with each of these mutations to better characterize their impact on the diagnosis and management of GBM.

## Materials and methods

### Institutional case selection

Ninety-one consecutive formalin-fixed, paraffin-embedded (FFPE) GBM specimens from 89 unique patients were submitted to Genomics and Pathology Services at Washington University in St. Louis (http://gps.wustl.edu) for testing between November 2012 and December 2015 (Supplemental Table 1). All cases were initially reviewed by board-certified neuropathologists according to the WHO 2007 guidelines for classification of tumors of the CNS^17^ and appropriate ancillary diagnostic work-up was done wherever necessary. Cases were re-classified according to WHO 2016 classification guidelines for the purpose of this study^18^.

**Table 1:** Differentially expressed gene count in comparisons of three molecularly distinct sub-types of glioblastoma.

Comparison	Up	Down
*BRAF* mutation versus *EGFR* mutation	305	156
*BRAF* mutation versus *IDH1* mutation	965	1078
*EGFR* mutation versus *IDH1* mutation	370	345

### Panel-based next-generation sequencing assays

The cases described in this study were derived from three separate versions of the same comprehensive cancer assay. The assay version is listed for each case in Supplemental Table 1, and the reportable genes for each assay version are listed in Supplemental Table 2. Ten genes were present on all three assay versions: *BRAF*, *CTNNB1*, *EGFR*, *IDH1*, *IDH2*, *KRAS*, *PDGFRA*, *PTEN*, *TP53*, and *WT1*.

### DNA preparation and next-generation sequencing

Next-generation sequencing was performed as previously described^19,20^. FFPE blocks were reviewed by board-certified neuropathologists to identify regions with sufficient neoplastic cellularity and tissue viability. Cores were punched from marked areas, and DNA was isolated using a Qiagen QIAamp DNeasy Blood and Tissue Kit (Qiagen, Hilden, Germany). DNA was sonicated with a Covaris Ultrasonicator (Woburn, MA), and assessed by an Agilent Bioanalyzer 2100 (Agilent, Santa Clara, CA). NGS libraries were prepared using commercially available kits (Supplemental Table 3). Whole genome shotgun libraries were enriched by hybridization capture using custom-designed oligonucleotide baits (Supplemental Table 3) corresponding to the genes included on each version of the assay. Enriched libraries were multiplexed and sequenced on the Illumina HiSeq2500 platform (Illumina, San Diego, CA) to generate 2 × 100 bp reads.

### Sample pre-processing and read alignment

Raw base call files (BCL files) were converted to fastq format using Cassava v1.8 (Illumina) and de-multiplexed using in-house scripts. Paired-end reads were aligned to the human reference assembly (UCSC hg19) using Novoalign (Novocraft Technologies, Selangor, Malaysia). Alignment files were converted to BAM format with samtools^21^, and duplicate reads were marked using Picard Tools MarkDuplicates (https://broadinstitute.github.io/picard/). Coverage metrics are provided in Supplemental Table 4.

### Variant prediction, annotation, and interpretation

Variant calling methods are outlined in Supplemental Table 3. Aligned read depth at each chromosomal position was determined using SAMtools mpileup^21,22^. Insertion and deletion (indel) variants ≤ 21 bp were called using Genome Analysis Toolkit’s (GATK^23^) Unified Genotyper with the following cutoffs: depth ≥ 50 reads, Fisher strand bias ≤ 75, and not adjacent to a homopolymer run > 7 bp. Single nucleotide variants (SNVs) were called using either Genome Analysis Toolkit’s (GATK) Unified Genotyper or VarScan2^24^ with the following cutoffs: depth ≥ 50 reads, base quality ≥ 20, mapping quality ≥ 30, Fisher strand bias ≤ 75, and variant allele fraction (VAF) > 3%. SNVs and indels present in reportable gene sets were subjected to annotation and clinical interpretation and were included in the patient’s medical record. Polymorphisms represented in public human genomic variation databases (gnomAD, ExAC, dbSNP, dbNSFP, NHLBI ESP) were discarded from this analysis. Remaining non-synonymous variants are listed in Supplemental Table 1. Variants were classified as pathogenic or likely pathogenic based on established criteria^25^.

### Fluorescence in situ hybridization

Formalin fixed paraffin embedded blocks were sectioned at 5 µm and routinely processed using the Vysis *EGFR*/CEP7 FISH probe kit (Abbott Molecular Inc., Glean Oaks, IL) and counterstained with DAPI II. Stained slides were viewed using a BX61 fluorescent microscope (Olympus, Melville, NY, USA) and photographed with a Jai Progressive Scan camera and CytoVision Imaging System (Leica Biosystems, Wetzlar, Germany). Two-hundred interphase nuclei were examined in each specimen. Polysomies and monosomies were determined based on centromere enumerating probes. Focal gains and losses were determined based on signal ratios from locus-specific versus centromere enumerating probes.

### Microarray-based gene expression studies

Primary, *IDHwt*, GBM specimens were selected from a TCGA dataset (Firehose Legacy, https://www.cbioportal.org/study?id=gbm_tcga) based on the presence of activating point mutations in *EGFR* and *IDH1*, and BRAF p.V600E mutations (Supplemental Table 5). CEL files from the selected cases were downloaded. Normalized signal values were generated using Expression Console v4.1.1 (Affymetrix, ThermoFisher Scientific, Waltham, MA) using the robust multichip average algorithm, and detection p-values were calculated using the MAS5 algorithm. Probe-sets were filtered based on the detection p-value; only those corresponding to coding genes were maintained in this analysis. Differentially expressed genes were determined using limma^26^ with fold change cutoff of ≥ 2 and a false-discovery rate (FDR) cutoff of ≤ 0.05.

### Biological theme enrichment analysis

CompBio software (https://www.percayai.com/) was used to identify biological themes that were enriched among the differentially expressed genes. This is accomplished with an automated Biological Knowledge Generation Engine (BKGE) that extracts abstracts from PubMed that reference the genes contained in the input list (i.e., the differentially expressed genes identified in the microarray analysis) and uses contextual language processing and a biological language dictionary to compute the statistical enrichment of biological concepts that co-occur with the input genes more frequently than would be expected by random chance. Related concepts derived from the list of differentially expressed genes are further clustered into higher-level themes. Scoring of concept and theme enrichment is accomplished using a multi-component function referred to as the Normalized Enrichment Score (NES). The first component utilizes an empirical p-value derived from several thousand random entity lists of a size that is comparable to the users input entity list to define the rarity of a given entity-concept event. The second component, effectively representing the fold enrichment, is based on the ratio of the concept enrichment score to the mean of that concept’s enrichment score across the set of randomized entity data. As such, the NES reflects both the rarity of the co-occurrence of the input genes with the concept as well as the degree of overall enrichment. Based on these empirical criteria, observed entity-concept scores above 10.0, 100.0, and 1,000.0 are labeled as moderate, marked, or high in level of enrichment above random. Themes scoring above 500.0, 1,000.0, and 5,000.0 are labeled similarly. In this study, the top 25 most enriched themes with scores > 1000.0 for each condition were subjected to manual annotation and further analysis (Supplemental Fig. 1).

**Figure 1:**
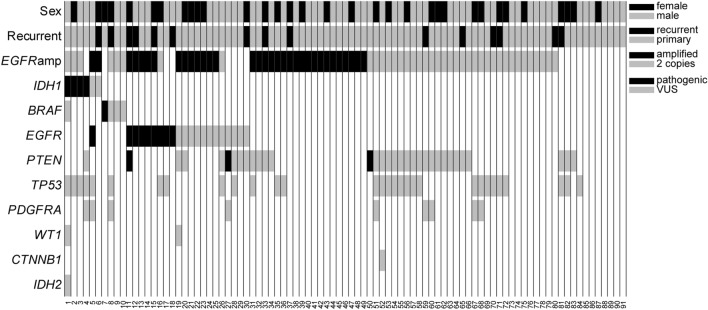
Mutations found in ninety-one specimens of glioblastoma. Ninety-one glioblastoma specimens from 89 unique patients were subjected to next-generation sequencing on a small gene panel used for routine clinical, diagnostic testing. Non-synonymous mutations are shown in nine genes that were included in all assay versions; common population polymorphism were excluded. Ancillary testing for *EGFR* amplification was performed via fluorescence in situ hybridization for 77 specimens as part of the diagnostic workup.

### Ethics approval

The institutional review board of Washington University School of Medicine determined that this retrospective study of de-identified data did not fit the definition of human subjects research; thus, no IRB protocol was required.

### Consent to participate

Not applicable.

### Consent for publication

All authors of this manuscript have directly participated in the planning, analysis, and/or editing of this study, and approved the publication of this work.

## Results

### Specimens and clinical data

Ninety-one GBM (WHO grade IV) specimens from 89 unique patients were submitted for targeted NGS between November 2012 and December 2015 (Supplemental Table 1). These specimens included 87 primary GBM (*IDH1 and IDH2* wild type, *IDHwt)* and four secondary GBM with mutations in *IDH1*. The male:female ratio among these patients was approximately 2:1 (56 M:33F). Patients ranged in age from 9 to 83 years at the age of biopsy (average 55.10 ± 14.97 years). Three pediatric/adolescent GBM (patients ≤ 18 years) were included in this cohort. One patient presented at age 12 with a GBM centered in the thalamic region and again at age 18 with a recurrence in the posterior fossa. The other two pediatric patients had been diagnosed with other cancers (desmoplastic infantile ganglioglioma and acute lymphoblastic leukemia) prior to the development of GBM.

### EGFR mutations

Twenty-one of the specimens in our dataset (23.1%) harbored point mutations in *EGFR* (Fig. 1, Supplemental Table 1)*.* Two specimens had EGFR p.G589V mutations; seven others had mutations in the extracellular domain (p.A289T, n = 1; p.A289D, n = 2; p.A289V, n = 4; Fig. 1, Supplemental Fig. 2). These mutations are common in GBM and are known to drive autophosphorylation and activation of EGFR in the absence of ligand^7,27^. The remaining 12 *EGFR* mutations were classified as variants of uncertain clinical significance (see methods for details); their impact on protein activity is unknown.

**Figure 2:**
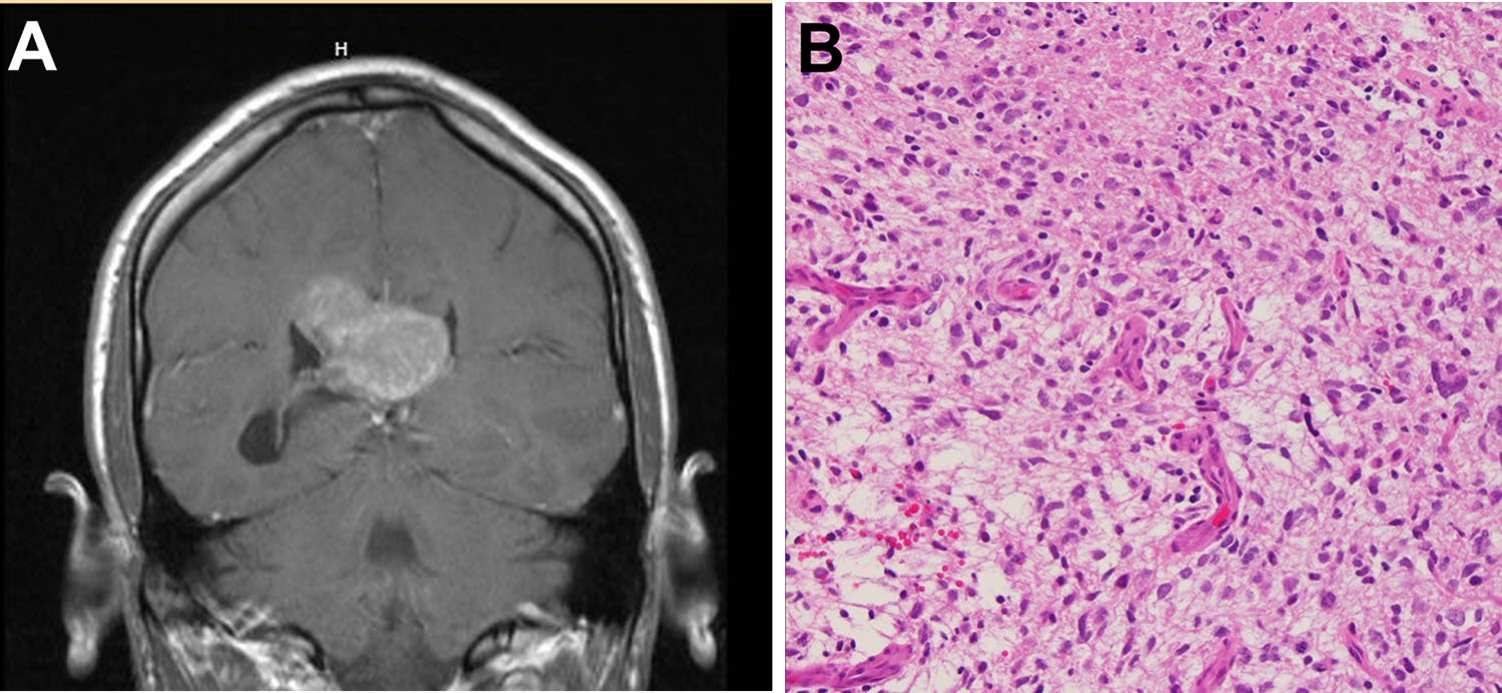
Magnetic resonance imaging and histological features of a representative *BRAF* mutated tumor. Patients with *BRAF* mutant GBMs were typically found adjacent to the ventricular system (**A**). *BRAF* mutated tumors did not show distinctive histomorphology compared to classical GBM (**B**).

Seventy-seven specimens were sent for analysis by FISH in addition to NGS. Thirty-three of these tested positive for *EGFR* gene amplification. Thirteen of the 33 had point mutations within the amplified gene (Fig. 1, Supplemental Table 1).

### BRAF mutations

Five specimens from our cohort had point mutations in *BRAF* (Fig. 1, Supplemental Table 1). One of these was the canonical p.V600E mutation (VAF 50.2%), which is known to increase kinase activity and drive oncogenesis^28^. While the other four mutations classified as variants of uncertain functional/clinical significance according to standard criteria (see methods for details), they were all located within the tyrosine kinase domain (Supplemental Fig. 2). In absence of a germline control (typically unavailable in the clinical setting), we take the VAF of the mutations (ranging from 10.0–26.0%) and their absence or rarity in databases of human genomic variation (e.g., gnomAD, see methods) as soft indications that they are somatic rather than germline polymorphisms. A search of the COSMIC database^29^ showed that the p.K483E and p.H574Q mutations found in tumors GBM_01 and GBM_08, respectively, have both been reported in the context of other cancers, most frequently in melanoma. The BRAF p.G596R mutation found in GBM_10 is classified as a class III BRAF mutation and is known to activate ERK signaling in a RAS-dependent manner despite impaired kinase activity^30^. The fifth BRAF mutation, found in patient GBM_09, was a frameshift present at the beginning of the kinase domain, and would thus be predicted to result in loss of function.

### Comparison of tumors with EGFR and BRAF mutations

As expected, *BRAF* and *EGFR* mutations were mutually exclusive in our cohort. *EGFR* gene amplifications and point mutations were only seen in primary GBMs, while one of the *BRAF* mutations was found alongside a gain-of-function mutation in *IDH1*. The three male and two female patients with SNVs in the *BRAF* gene were, on average, younger than the 21 patients with *EGFR* SNVs (mean, 41.82 ± 15.94 and 53.19 ± 11.33 years, respectively).

The location of the tumor within the brain was known for 87 of the 91 specimens analyzed. Tumors were found adjacent to the ventricular system in 3 of the 5 patients with *BRAF* mutations (Fig. 2A, Supplemental Table 1). Of the two remaining *BRAF*-mutant tumors, one was found in the frontal lobe involving the precentral gyrus and the location of the other was unknown. In comparison, tumors without *BRAF* mutations showed a range of locations, most commonly involving the frontal and temporal lobes and, less frequently, the parietal lobes, occipital lobes or midline structures (Supplemental Table 1).

The *BRAF*-mutant tumors in this cohort set did not exhibit distinctive histomorphology (Fig. 2B). None were characterized as epithelioid GBM.

### Impact of BRAF and EGFR mutations on survival

**Figure 3:**
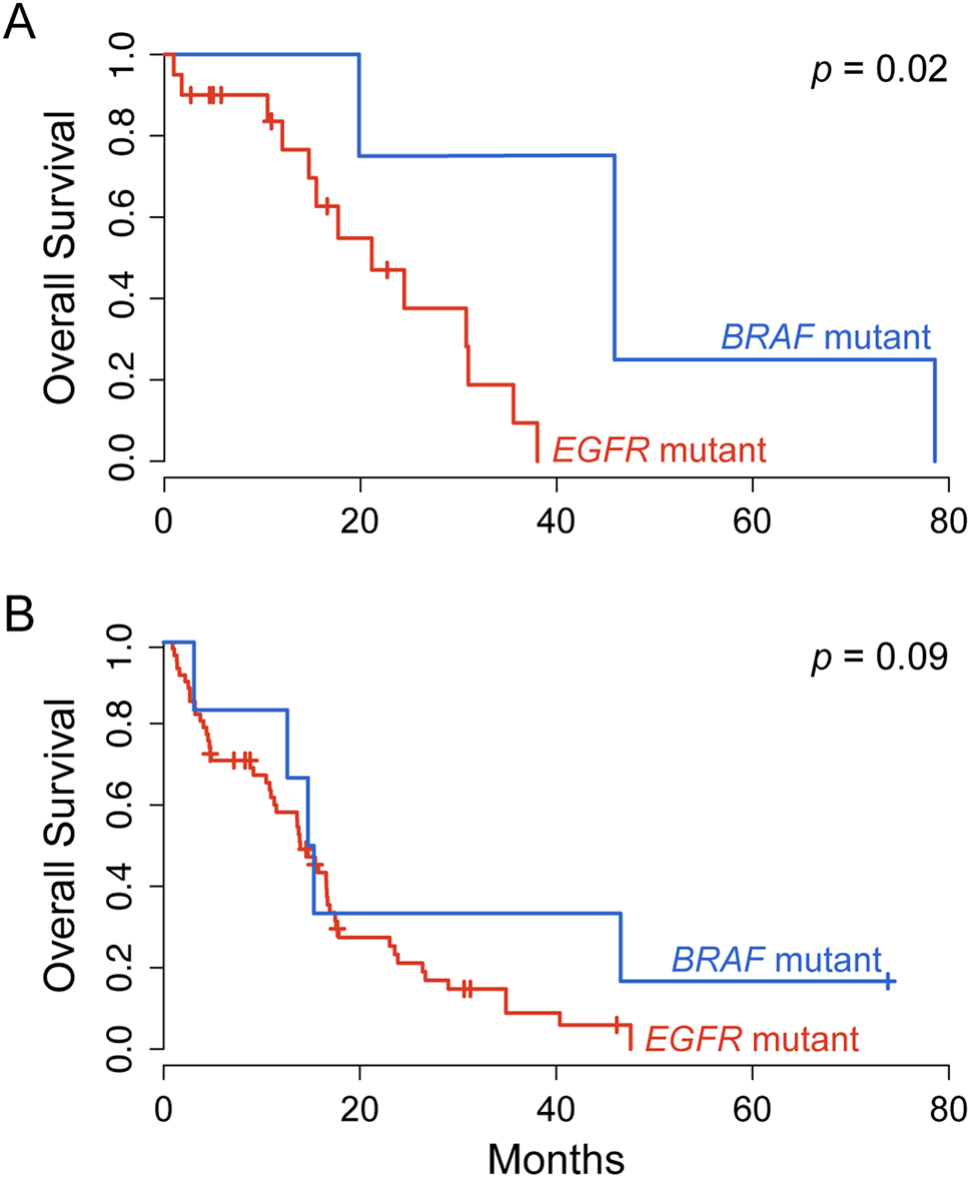
The impact of *BRAF* versus *EGFR* mutations on overall survival in glioblastoma. Kaplan–Meier plots indicate the difference in survival between *BRAF* and *EGFR* mutated tumors in our in-house cohort (**A**) and a dataset from TCGA (**B**). Considering only *IDH1* wild-type tumors, the average overall survival of three patients with putative activating *BRAF* mutations was longer than that of 20 patients with *EGFR* mutations from the in-house cohort (average overall survival of 48.01 ± 24.42 months versus 16.13 ± 11.46 months, respectively; log-rank *p* = 0.02). One patient with an activating *IDH1* mutation and another with a *BRAF* frameshift mutation (likely loss-of-function) were omitted from this analysis. Likewise, the average overall survival of 6 patients with gain-of-function *BRAF* mutations was longer compared to 77 patients with gain-of-function *EGFR* mutations in a cohort from TCGA (average overall survival of 27.68 ± 26.95 and 14.19 ± 11.28 months, respectively; log-rank *p* = 0.09).

Survival data was available for 85 of 91 patients. Kaplan–Meier curves were calculated to compare the outcomes of tumors with *EGFR* versus *BRAF* mutations (Fig. 3A). Excluding GBM_01 (a secondary GMB) and GBM_09 (harboring the likely-inactivating frameshift mutation), the average overall survival of three patients with *BRAF-*mutated tumors was longer than that of patients with *EGFR-*mutated tumors (average of 48.01 ± 24.42 months versus 16.13 ± 11.46 months, respectively; log-rank p-value = 0.02). These findings should be considered as preliminary given the small size of the *BRAF-*mutated cohort.

We leveraged a TCGA dataset (Firehose Legacy, https://www.cbioportal.org/study?id=gbm_tcga) to further explore the implications of *BRAF* mutations on survival outcomes. Mutation data was provided for 290 of the 619 specimens included in this cohort. Six of the 290 profiled tumors harbored gain-of-function mutations in *BRAF* (5 p.V600E, 1 p.G596D) while 77 had gain-of-function mutations in *EGFR*. Consistent with our in-house dataset, primary GBM patients with *BRAF-*mutated tumors survived somewhat longer than patients with *EGFR*-mutated tumors (Fig. 3B; mean, 27.68 ± 26.95 and 14.19 ± 11.28 months, respectively; log-rank p-value = 0.09).

### Differential gene expression in IDHwt-GBM with BRAF and EGFR mutations

Microarray data from the TCGA cohort described above was used to assess gene expression profiles in GBM with distinct mutation signatures (Table 1). mRNA expression data was only available for three *IDHwt* tumors with BRAF p.V600E mutations. These were compared to expression data from 12 randomly selected *IDHwt* tumors with gain-of-function *EGFR* mutations (individuals described in Supplemental Table 5). We included a third group of tumors with *IDH1* mutations to help determine the relative directional change of the observed expression differences. Counts of genes considered differentially expressed across the three comparisons are listed in Table 1.

**Figure 4:**
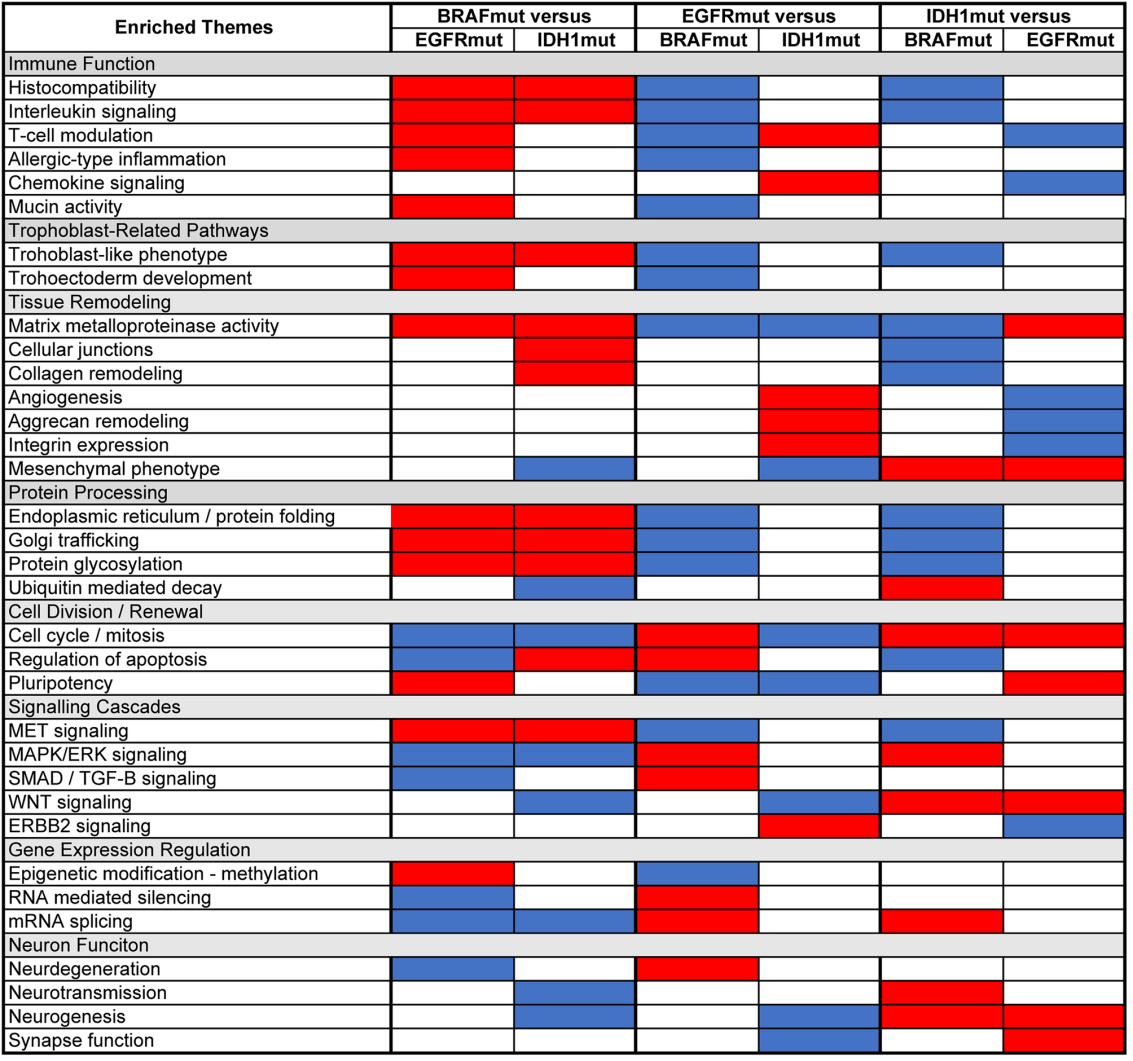
Biological concepts and themes enriched among genes differentially expressed between tumors gain-of-function mutation in *BRAF*, *EGFR*, or *IDH1.* A pseudo-heatmap was constructed from the comparison of biological themes associated with the differentially expressed genes identified from a microarray based study of gene expression profiles among glioblastoma with gain-of-function mutations in *BRAF*, *EGFR* and *IDH1*. Related biological themes that were associated with each list of differentially expressed genes (illustrated in Supplemental Fig. 1) were grouped into higher-level clusters to facilitate comparison between the three subgroups. Red indicates increased expression; blue indicates decreased expression.

Biological theme enrichment analysis indicated that tumors with *BRAF* gain-of-function mutations show increased expression of genes implicated in MET pathway signaling, protein processing, immune function, and invasion, among other features that might be expected in a tissue exhibiting intense metabolic activity and cell growth/division (Fig. 4, Supplemental Fig. 3, Supplemental Video 1). Of note, we observed elevated expression of several genes typically thought to be selectively expressed in trophoblasts, including a complement of pregnancy-specific glycoproteins (PSGs) and HLA-G (Supplemental Fig. 4).

Compared to the other two groups, tumors with *IDH1* mutations showed a strong stem-like gene expression profile (Fig. 4). This included increased expression of genes related to cell cycle / mitosis, transformation to a mesenchymal phenotype, and Wnt signaling, as well genes involved in neurogenesis.

Little overlap was seen in the comparison of tumors with *EGFR* gain-of-function mutations to tumors with *BRAF* versus *IDH1* mutations (Fig. 4).

Compared to tumors with *BRAF* mutations, *EGFR-*mutant tumors showed elevated expression of genes involved in cell cycle function, regulation of apoptosis, and diverse signaling cascades. They also showed increased expression of gene in the *ERBB2* signaling cascade relative to tumors with *IDH1* mutations.

## Discussion

In this study, we combined data from an in-house cohort of 89 patients with data from TCGA to explore the clinical significance of gain-of-function mutations in *BRAF* versus *EGFR*. As previously shown, activating mutations in these genes were mutually exclusive, suggesting that the two subtypes may arise through a distinct mechanism that leads to different pathological and clinical features. Although *BRAF*-mutant GBM are rare, patients with *BRAF*-mutant tumors were, on average, younger and survived longer relative to those with *EGFR*-mutant tumors. Despite similar histomorphology, *BRAF-*mutant tumors often arose centrally in the brain, adjacent to the ventricular system. Microarray studies indicated that *BRAF*-mutant tumors have a unique mRNA expression profile compared to *EGFR-* and *IDH1*-mutant tumors, with upregulation of genes related to immune tolerance and invasion, suggesting a unique mechanism for tumorigenesis in this subset of GBM.

The aim of this study was not to describe the genomic landscape of GBM tumors; this has been reported elsewhere^4,10^. Relatively few genes were sequenced and analyzed in our in-house cohort, as the NGS panel was selected for clinical and diagnostic purposes rather than research, and most clinical NGS panels are limited in scope to minimize cost and analytical time. Furthermore, the presently described assay was not suitable for the detection of gene fusions, some of which are known to activate BRAF and the MAPK pathway^31^. The size and scope of the clinical NGS panel remains a limitation of this work though our results indicate that the in-house cohort was similar to those described in the literature, particularly with respect to the frequency of *EGFR* and *BRAF* mutations^4,32,33^. Further work will be needed to fully characterize the genomic profile of these two groups.

Patients with GBM harboring *BRAF* mutations presented earlier in life compared to those patients whose tumors had *EGFR* mutations. In other studies, BRAF p.V600E mutations have been associated with epithelioid features in GBM^34^. BRAF p.V600E mutations are common (~ 50% frequency) in epithelioid GBM, a sub-type that often occurs in young adults and children^35^. In contrast, only one of our *BRAF* mutated tumors was found in a pediatric patient (GBM_08, Supplemental Table 1) and none were characterized as the epithelioid subtype. The histological appearance was conventional in all *BRAF*-mutated specimens from our in-house cohort (Fig. 2).

While the histological appearance of the *BRAF*-mutant tumors included in this study was unremarkable, we did note that three of the four patients with available clinical data had *BRAF-*mutant tumors located adjacent to the ventricular system. Other studies have indicated that overall survival is decreased for GBM bordering the lateral ventricles^36^. Despite the small cohort size, our analysis suggested that patients with *BRAF*-mutant tumors may survive longer compared to tumors with *EGFR* mutations, which were most often found in the cerebral lobes (Supplemental Table 1). This difference in overall survival should be considered as a preliminary finding, as statistical significance was not achieved (likely due to the paucity of *BRAF*-mutant tumors). Furthermore, the rarity of *BRAF*-mutated tumors precluded multi-variate analyses that could help elucidate the role of other impactful features such as age or sex. Indeed, patient age may be a key factor in survival since patients with *BRAF* mutant tumors were, on average, younger than patients with *EGFR* mutated tumors. Nonetheless, the potential difference in outcomes is interesting, particularly in the absence of targeted treatments (e.g., FDA-approved BRAF inhibitors like dabrafenib and vemurafenib)^37–39^.

The most striking differences between *BRAF* mutant tumors compared to other groups of GBM were seen at the level of gene expression. *BRAF*-mutant tumors showed a unique mRNA expression profile, with increased expression of genes involved in immune modulation, including the *HLA-G* gene. HLA-G is a non-classical human leukocyte antigen class I molecule that functions in fetal-maternal immune tolerance^40^. It is possible that this profile serves a similar function in tumors, suppressing the anti-tumor response through interaction with inhibitory receptors on immune cells^41^. These findings could have implications for traditional checkpoint inhibition therapy (anti-CTLA-4 or anti-PD-1), in that the expression of HLA-G could serve as an alternate route for immune evasion in *BRAF* mutated GBM.

A host of PSGs were also upregulated in *BRAF*-mutated tumors, which resulted in a trophoblast-like gene expression profile. PSGs are a collection of secreted glycoproteins abundant in maternal blood during pregnancy. Like HLA-G, they play an important role in embryonic development^42^, and have been associated with upregulated expression in various cancers^43–46^. For example, studies have shown that PSG9 serves as a driver of angiogenesis in colorectal and hepatocellular cancers^47,48^. There are marked similarities between tumor invasion and trophoblast implantation^49,50^, so our results may suggest that *BRAF*-mutant GBM may co-opt trophoblast-like properties to facilitate their growth and spread.

While we have presented evidence that *EGFR-* and *BRAF-*mutated classical GBM are biologically distinct, we cannot state with certainty whether these differences are directly caused by the mutations identified in these two genes. It is possible that these mutations represent markers of particular tumor types, rather than the direct cause of their distinction. This is particularly true of *EGFR* mutations, which are known to correlate with various other impactful alterations (e.g., *TERT* promoter mutations, gain of chromosome 7, loss of chromosome 10, etc.)^10^. It is also possible that *BRAF-*mutated tumors are representative of a larger sub-type of non-*EGFR*-mutated GBM rather than a unique entity of their own. Regardless, the unique features of these two groups warrant further exploration.

## Conclusions

*BRAF*-mutant GBM are very rare, especially in adult populations; however, differences in tumor location, survival rates, and global gene expression profiles set this set of tumors apart from other GBM. Striking differences were seen in comparison to tumors with *EGFR* gain-of-function mutations, another member of the same signaling cascade. Although this cohort was modest in size, *BRAF*-mutant GBM constituted a unique subgroup with potential implications for tumor biology and treatment.

## Supplementary Information

Supplementary Figure 1.

Supplementary Figure 2.

Supplementary Figure 3.

Supplementary Figure 4.

Supplementary Legends.

Supplementary Video 1.

Supplementary Table 1.

Supplementary Table 2.

Supplementary Table 3.

Supplementary Table 4.

Supplementary Table 5.

## Data Availability

The clinical data generated from this study are available in the associated supplemental materials.
