# Supplementary figures and images for: BRAF mutations may identify a clinically distinct subset of glioblastoma
Source: Sci Rep. 2021 Oct 8;11:19999. doi: 10.1038/s41598-021-99278-w (PMC8501013; doi:10.1038/s41598-021-99278-w)

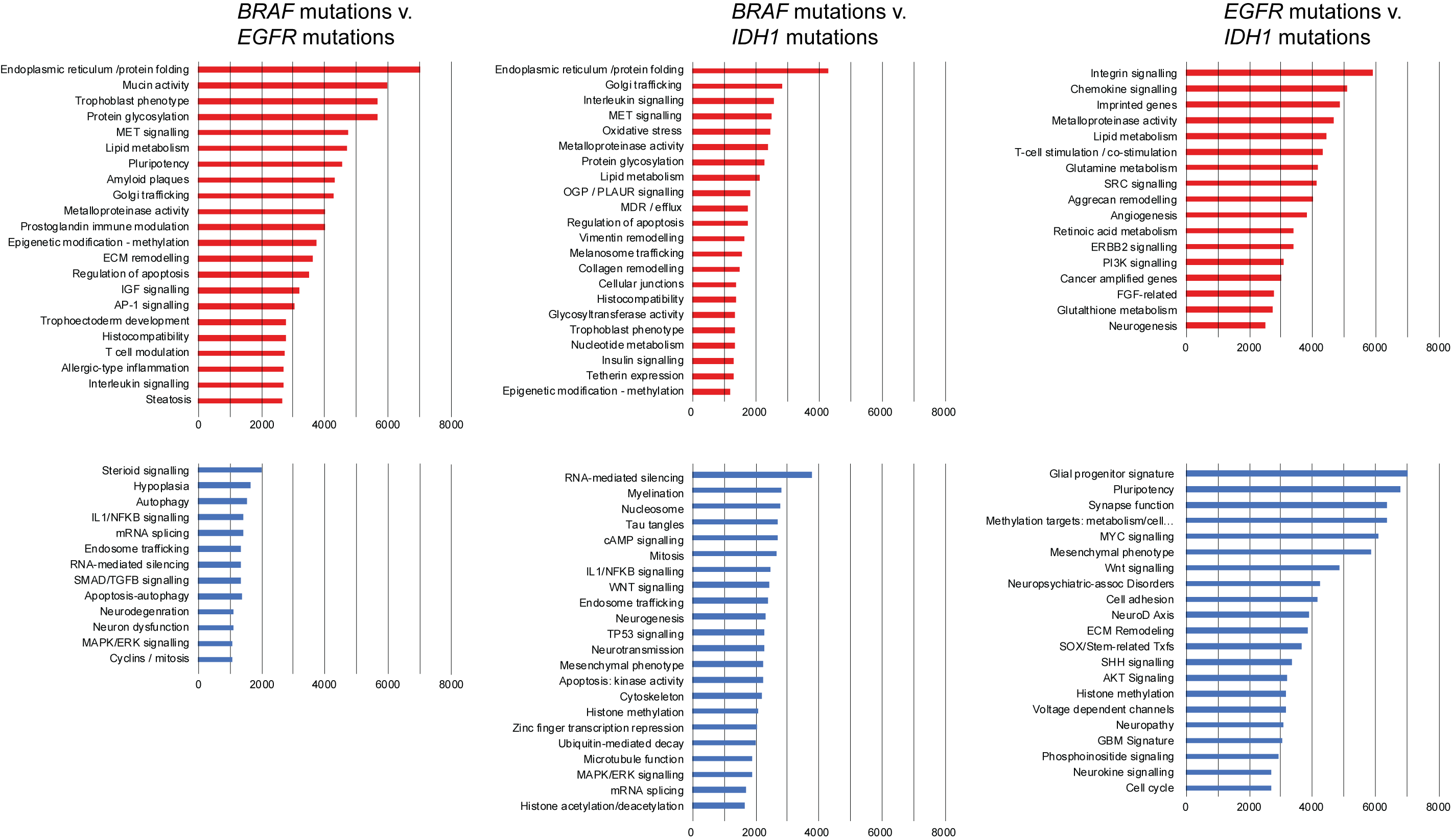

Supplement: Supplementary file 1 — Supplementary Figure 1. [file 41598_2021_99278_MOESM1_ESM.tif]

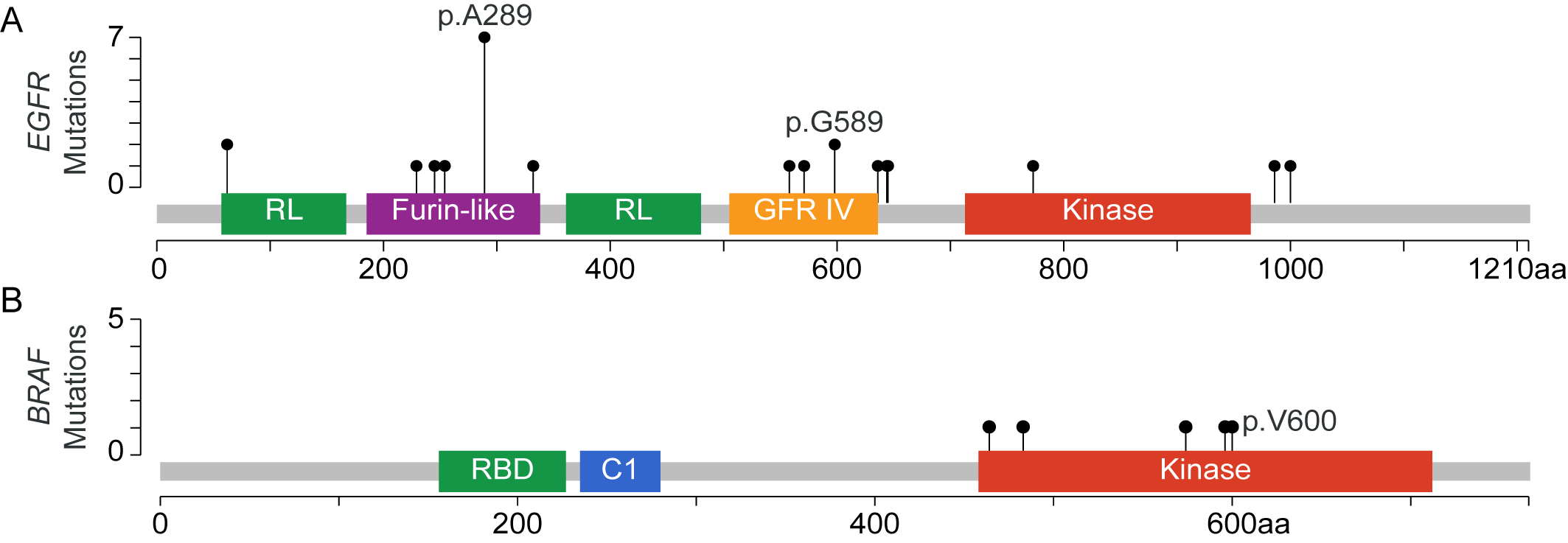

Supplement: Supplementary file 2 — Supplementary Figure 2. [file 41598_2021_99278_MOESM2_ESM.tif]

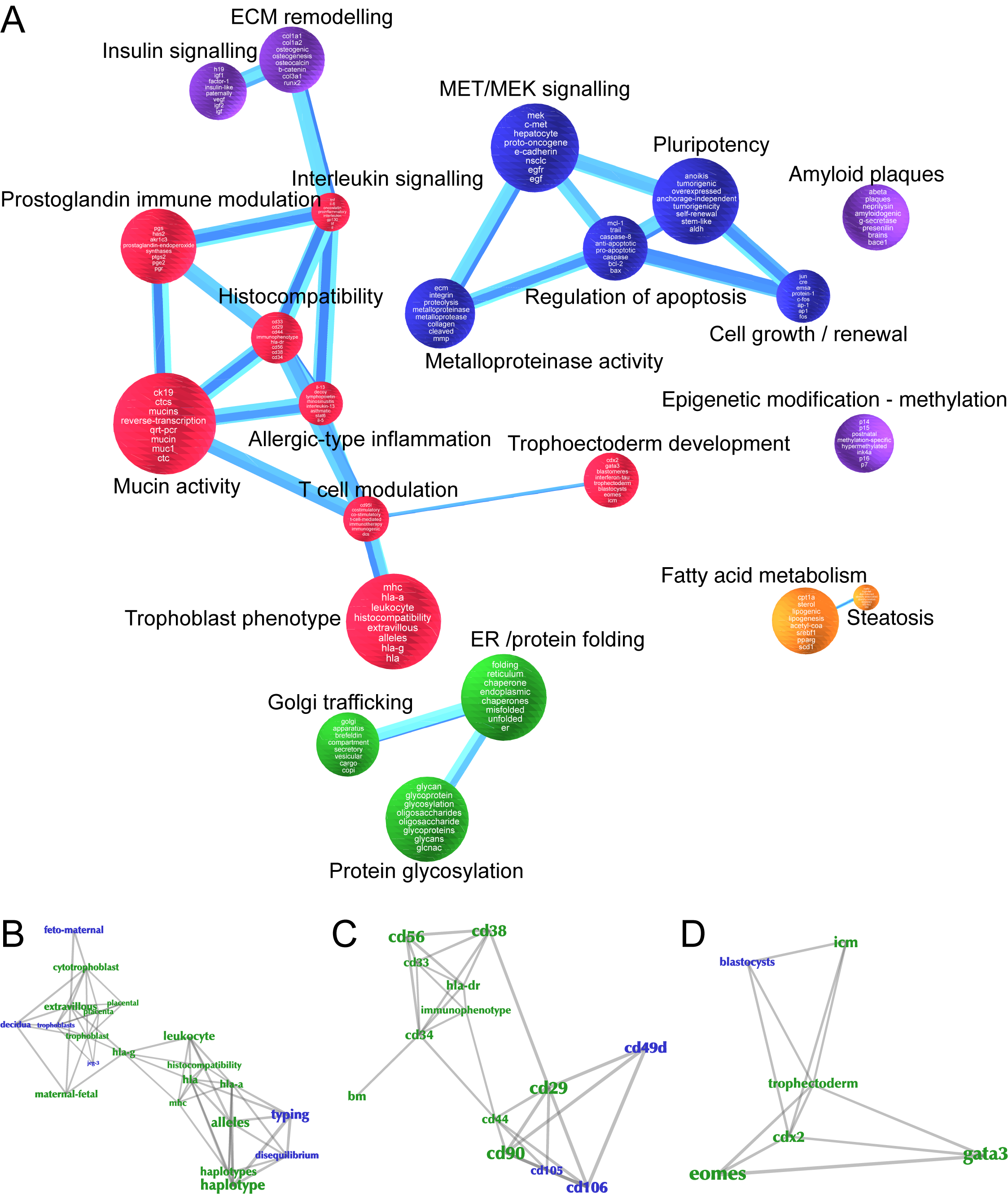

Supplement: Supplementary file 3 — Supplementary Figure 3. [file 41598_2021_99278_MOESM3_ESM.tif]

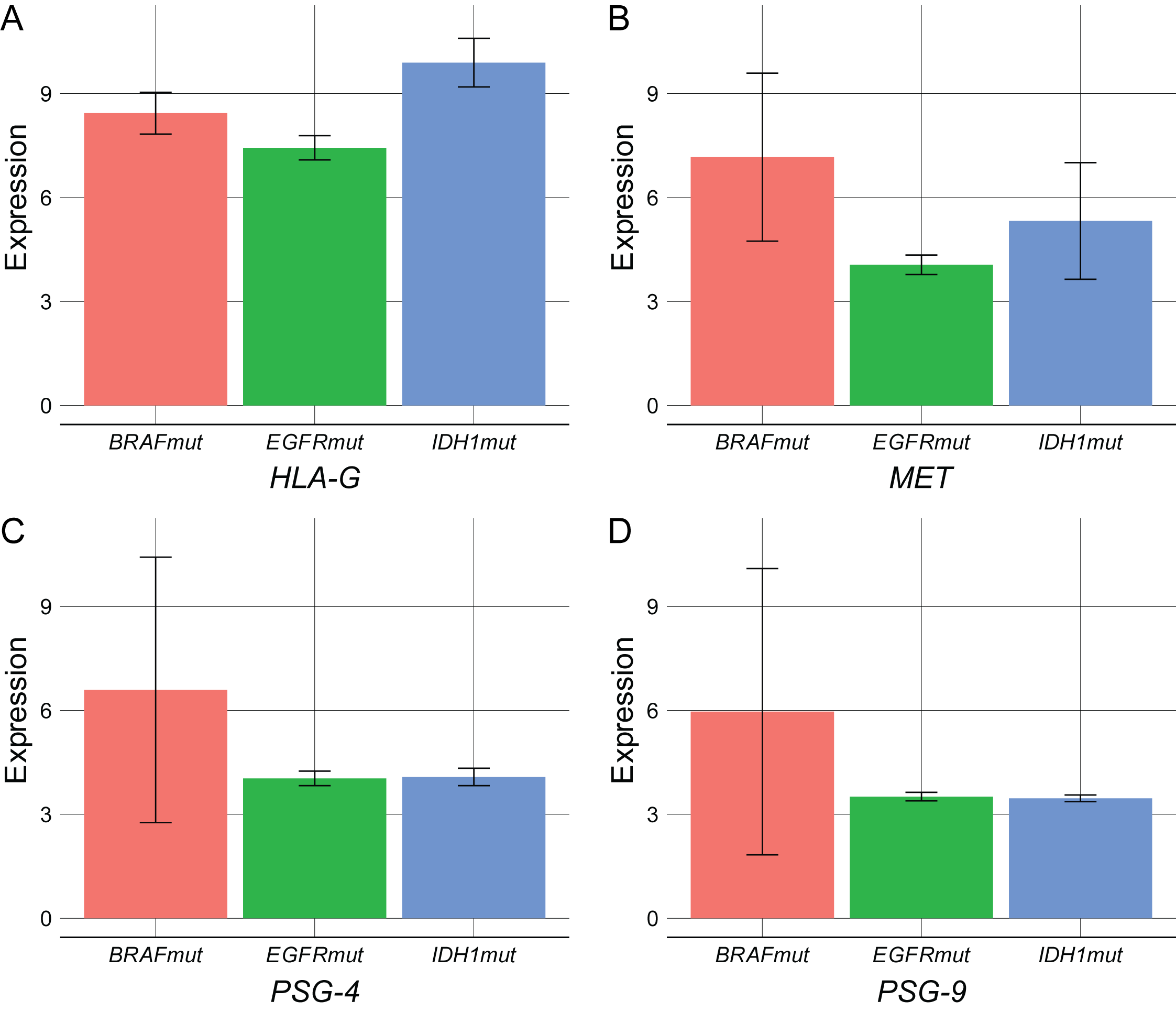

Supplement: Supplementary file 4 — Supplementary Figure 4. [file 41598_2021_99278_MOESM4_ESM.tif]
