# Supplementary material for: BRAF mutations may identify a clinically distinct subset of glioblastoma
Source: Sci Rep. 2021 Oct 8;11:19999. doi: 10.1038/s41598-021-99278-w (PMC8501013; doi:10.1038/s41598-021-99278-w)
Supplement: Supplementary file 5 — Supplementary Legends. [file 41598_2021_99278_MOESM5_ESM.docx]

**SUPPLEMENTAL FILES**

**Supplemental Figure 1. Enriched biological themes associated genes that are differentially expressed between tumors with mutations in *BRAF, EGFR,* and *IDH1***

Enriched biological themes were generated using CompBio software from lists of genes that were found to be differentially expressed between tumors with mutations in (A) *BRAF* versus *EGFR*, (B) *BRAF* versus *IDH1*, and (C) *EGFR* versus *IDH1*. The data from this analysis were used to generate the pseudo-heatmap shown in Figure 5. Red indicates increased expression while blue indicates decreased expression.

**Supplemental Figure 2. Missense mutations in *EGFR* and *BRAF***

Twenty-one missense mutations in *EGFR* were identified among our 91 specimen cohort (A). These included nine mutations with known gain-of-function effects: 7 at the p.A289 position and two at the p.G589 position. Conversely, only five missense mutation were found in *BRAF* (B). One of these was the p.V600E mutation. The other *BRAF* variants, while classified as variance of uncertain significance, fell within the kinase domain.

**Supplemental Figure 3. Enriched theme- and concept- level views from CompBio analysis of genes with upregulated expression in *BRAF* mutated tumors in comparison to *EGFR* mutated tumors.**

Spheres represent biological themes that emerged computationally, in an unsupervised manner, from the analysis of a list of genes that were upregulated in *BRAF* mutated tumors compared to *EGFR* mutated tumors. The blue lines that connect the spheres represent the links between the themes through their shared genes. Biological theme (sphere) proximity is determined by the degree of relationship between their concepts as computed through the contextual language analysis. The lower 3 panels illustrate individual concepts that comprise three key biological themes: trophoblast phenotype (B), histocompatibility (C), and trophoectoderm development (D).

**Supplemental Figure 4. Expression of genes upregulated in tumors with mutations in *BRAF* compared to *EGFR* and *IDH1***

Log-2 signal intensity values ± standard deviation were plotted for four genes found to be significantly differentially expressed between tumors with gain-of-function mutations in *BRAF* versus *EGFR*. Tumors with *IDH1* mutations were included for context.

**Supplemental Video 1. Animated depiction of enriched themes from CompBio analysis of genes with upregulated expression in *BRAF* mutated tumors in comparison to *EGFR* mutated tumors.**

Spheres represent biological themes that emerged from the analysis of a list of genes that were upregulated in *BRAF* mutated tumors compared to *EGFR* mutated tumors. The blue lines that connect the spheres represent the links between the themes (i.e., genes shared between the themes). The closer the spheres, the more closely the themes are related.

**Supplemental Table 1. Clinical, pathologic, and molecular data gathered from 91 specimens of GBM**

**Supplemental Table 2. Genes included in three versions of the Comprehensive Cancer Gene Set assay**

**Supplemental Table 3. Methodological details of three versions of the Comprehensive Cancer Gene Set assay**

**Supplemental Table 4. Depth of coverage of assay capture space**

**Supplemental Table 5. Metadata describing TCGA patients included in microarray gene expression study**
